# Exploring the Role of a Noninvasive Audio-Based Therapeutic System for Pain and Symptom Relief: Single-Arm Exploratory Pilot Study

**DOI:** 10.2196/96015

**Published:** 2026-09-02

**Authors:** Lea Sacca, Isabella Abraham, Panagiota Kitsantas, Robert Sewak, Chengwu Yang, Timothy D Dye, Charles H Hennekens

**Affiliations:** 1 Department of Population Health Charles E Schmidt College of Medicine Florida Atlantic University Boca Raton, FL United States; 2 Department of Health Administration and Policy George Mason University Fairfax, VA United States; 3 Florida Institute of Technology Melbourne, FL United States; 4 Department of Medicine Charles E Schmidt College of Medicine Florida Atlantic University Boca Raton, FL United States

**Keywords:** non-invasive sound therapy, chronic pain, symptom relief, audio-based, Transdermal Acoustic Pain Suppression intervention

## Abstract

**Background:**

In the United States and worldwide, chronic pain affects a vast number of people and is one of the leading reasons adults seek medical care. In the United States, 24.3% of adults reported experiencing chronic pain in the prior 3 months in 2023. Chronic pain is defined as pain that lasts ≥3 months, significantly disrupting one’s daily functioning and quality of life. Chronic pain can also be accompanied by other conditions, including anxiety and depression. Health care providers should be aware of several limitations in their current treatment modalities. Although opioid analgesics are used for moderate to severe pain management, they cause many serious adverse effects, including sedation, respiratory depression, constipation, and a high risk of dependence and addiction. Other pain medications, such as nonopioid analgesics, including nonsteroidal anti-inflammatory drugs, adjuvant analgesics, and corticosteroids, also cause a range of side effects and organ toxicity.

**Objective:**

We conducted a single-arm exploratory pilot study to explore the potential role of a noninvasive, transdermal, audio-based therapeutic system among 24 police professionals aged 21 to 65 years with chronic pain and/or associated conditions, including migraine, sleep disorders, stress, anxiety, and conventional headaches.

**Methods:**

In this STROBE (Strengthening the Reporting of Observational Studies in Epidemiology)–guided pilot study, we explored the feasibility and perceived effectiveness of a self-administered intervention based on the Transdermal Acoustic Pain Suppression (TAPS) system among police personnel in West Palm Beach, Florida. Participants completed a flexible 30-day protocol integrated into their work schedules, with data collection spanning 5 months due to operational constraints. We used percentages as measures of effect and the chi-square test and Fisher exact test for significance testing. All *P* values were 2-sided, with a level of significance of .05.

**Results:**

In this small pilot study, following TAPS therapy, more than one-third (n=9, 37.5%) of participants experienced improvement in their condition, and half (n=12, 50%) reported reduced symptoms, including notable pain reduction.

**Conclusions:**

Although the findings from this small pilot study are preliminary, we believe they support the rationale for larger analytic studies designed a priori to test the hypothesis.

## Introduction

In the United States and worldwide, chronic pain affects a vast number of people and is one of the leading reasons adults seek medical care. In the United States, 24.3% of adults reported experiencing chronic pain in the past 3 months in 2023 [1]. Chronic pain is defined as pain that lasts ≥3 months, significantly disrupting one’s daily functioning and quality of life [2]. Chronic pain can also be accompanied by other conditions, including anxiety and depression. It has been estimated that 5% of US adults living with chronic pain regularly report feelings of depression, while 12% report feelings of worry, nervousness, and anxiety [3]. Other conditions associated with chronic pain are headaches and sleep disorders, which can be similarly debilitating. In 2021, 4.3% of adults living with chronic pain reported experiencing headaches or migraines in the past 3 months [4]. In 2020, 14.5% of this population had difficulty falling asleep on most days or every day in the past 30 days [5]. These conditions not only impact the affected individuals but also pose significant public health and socioeconomic burdens. In a cross-sectional study, the economic costs of chronic pain in the United States were estimated to be US $722.8 billion, including US $530.6 billion in medical care costs and US $192.2 billion in lost work productivity [6].

Health care providers should be aware of several limitations in their current treatment modalities [7]. Although opioid analgesics are used for moderate to severe pain management, they cause many serious adverse effects, including sedation, respiratory depression, constipation, and a high risk of dependence and addiction [7]. Other pain medications, such as nonopioid analgesics, including nonsteroidal anti-inflammatory drugs, adjuvant analgesics, and corticosteroids, also cause a range of side effects and organ toxicity [7]. Medication choices are also limited by a patient’s specific characteristics, such as the nature and severity of their pain, comorbidities, age-related physiological changes, and drug interactions [7]. Another limitation is accessibility to these treatments [8]. Among respondents to a survey conducted by the American Chronic Pain Association, more than half experienced difficulties in receiving pain care and accessing pain medications, which led to consequences such as unmanaged pain, psychological distress, and suicidal ideation [8]. Due, in part, to these challenges, there is a growing interest in noninvasive and nonpharmacological interventions for pain management [9]. Furthermore, there is increasing evidence that sound- and music-based therapies are effective in reducing the perception of pain and distress while being inexpensive, safe, and noninvasive [10,11].

Sound and frequency stimulation may benefit physiological and psychological outcomes, particularly autonomic regulation, stress responses, and pain perception [10]. The autonomic nervous system (ANS) usually maintains a homeostatic balance between the sympathetic and parasympathetic nervous systems [10]. In addition, mental or physical stress from chronic pain causes greater sympathetic activity [10]. Music-based interventions lower heart rate and blood pressure while increasing heart rate variability, indicating enhanced parasympathetic nervous system activity and resulting in rebalancing of the ANS [10]. This effect may reduce pain perception and stress hormones such as cortisol [10]. Additionally, cortical and subcortical areas that are involved in pain processing are also activated when exposed to music-related stimuli [10]. These shared receptors include those of β-endorphins, oxytocin, dopamine, and serotonin, with oxytocin having immune-regulatory and anti-inflammatory effects that reduce pain perception, as well as calming and anxiolytic effects [10]. Another mechanism affecting pain perception is oscillatory brain activity, defined as rhythmic patterns of synchronized neural activity of varying frequencies [10]. Music comprises multiple sound frequencies, which affect this oscillatory activity and, thereby, pain perception [10].

Building on these principles, the Transdermal Acoustic Pain Suppression (TAPS) system introduced by Scientific Sound Works, LLC (SSW), is an innovative, audio-based, noninvasive therapeutic system designed to use sound therapy for pain modulation. The TAPS system uses multiwaveform sequencing technology, which differs from traditional sound therapies by delivering dynamically layered and sequenced frequencies that can target multiple neural oscillation patterns simultaneously, rather than relying on a single tone or static frequency. Similar noninvasive technologies that use multiple patterned frequencies have been shown to reduce pain, such as the M-Stim device, a thermomechanical stimulation system that delivers multifrequency mechanical vibration [12,13]. In a randomized crossover study of individuals with fibromyalgia, patients assigned to presleep alpha-frequency entrainment delivered via flickering light or binaural beats had reductions in pain and improvements in sleep quality [14].

Data are sparse concerning the effects of the TAPS system on chronic pain management. Such data could illuminate individual experiences, symptom changes, and potential clinical applications of the TAPS system. On the basis of these considerations, we conducted an exploratory single-arm pilot study to explore the potential role of the TAPS system using a noninvasive transdermal audio-based therapeutic device (a commercially available over-the-counter bone conduction transducer), in providing relief to police professionals aged 21 to 65 years with chronic pain and/or associated conditions, including migraine, sleep disorders, stress, anxiety, and conventional headaches.

## Methods

### Study Design

In this pilot study, we explored the implementation, acceptability, and participant-reported experiences associated with a self-administered TAPS intervention among police personnel. This small case series was intended to generate preliminary information to decide whether they inform the design of future analytic studies designed a priori to test the hypotheses. The reporting of this study was guided by the STROBE (Strengthening the Reporting of Observational Studies in Epidemiology) guidelines.

### Ethical Considerations

The study protocol was reviewed and approved by the institutional review board of Florida Atlantic University (IRB#2510332). All participants provided informed consent before participation.

### Study Setting

The study was conducted within the West Palm Beach Police Department in West Palm Beach, Florida. Study activities occurred primarily in the designated patrol division briefing room and the office of the departmental liaison. The occupational setting required flexibility because participants’ availability varied according to duty rotations, work schedules, illness, vacation, reassignment, and other operational responsibilities.

Each participant was assigned a 30-day intervention period. However, completion of study activities across the full sample required slightly more than 5 months because participants initiated and completed their individual study periods at different times and because of operational, scheduling, personnel, and minor technical disruptions. Thus, the 5-month period reflects the overall duration of study implementation across participants rather than a 5-month intervention exposure for each participant.

### Participant Eligibility

Participants were police personnel aged 21 to 65 years who were recruited from the participating police department. The study enrolled 24 participants. Because the pilot study included participants with potentially heterogeneous baseline symptoms and health conditions, it was not designed to estimate condition-specific treatment effects.

Patient perceptions were assessed using an investigator-developed questionnaire, which collected participant-reported conditions, the likelihood of trying audio-based therapy (not at all likely, somewhat likely, likely, or very likely), the perceived effectiveness of the intervention (significantly worse, slightly worse, no change, slightly better, or significantly better), perceived changes in symptom frequency and/or intensity (reduction in frequency, reduction in intensity, reduction in both, no change, or unsure), overall condition change (reduction, no change, increase, or unsure), and overall satisfaction (very dissatisfied, dissatisfied, neutral, satisfied, or very satisfied). These measures were not predefined using standardized or validated clinical criteria but were based solely on participants’ self-reported perceptions collected through this questionnaire. Accordingly, these outcomes should be interpreted as subjective participant perceptions rather than objective measures of clinical improvement or treatment efficacy.

### Recruitment and Informed Consent

**Figure 1:**
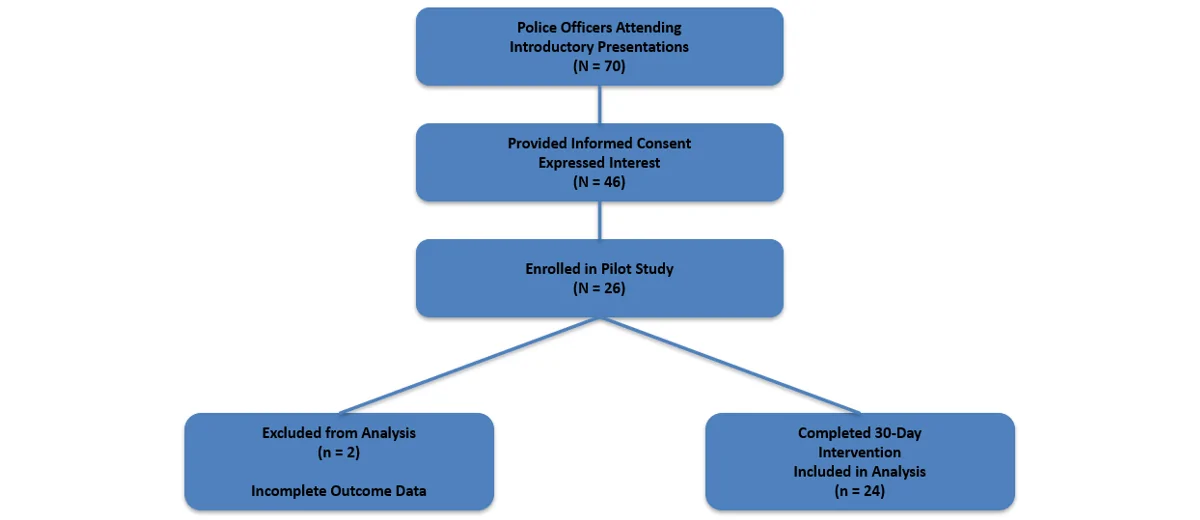
Participant flow diagram.

Recruitment began with introductory presentations attended by approximately 70 police officers, of whom 46 (65.7%) expressed interest and provided informed consent. Of these, 26 (37.1%) participants enrolled in the study, and 24 (34.3%) completed the 30-day intervention and were included in the final analyses. The 2 (2.9%) excluded participants were not included in the final dataset because of missing data on most questionnaire items (Figure 1). Each participant completed a flexible 30-day self-administered intervention period, although implementation across the full sample varied slightly because of duty rotations, scheduling conflicts, illness, vacation, personnel reassignment, and other operational demands. Minor technical and operational issues were encountered during implementation but did not prevent study completion. Because the intervention was intentionally designed to accommodate participants’ work schedules, sessions were completed on an as-needed basis or at participants’ convenience.

### Intervention and Device Description

The intervention explored in this pilot study was the TAPS system, which uses a device to transmit a low-frequency audio signal to the area where pain is perceived. The user positions the device at the affected site, where it delivers a scientifically engineered and specifically correlated audio signal via Bluetooth from a smartphone, computer, tablet, or similar device. This signal interacts with the pain signal, thereby disrupting and suppressing its transmission. The device’s triple-action mechanism, combining sound energy, vibration, and heat, further enhances its effectiveness by interfering with the brain’s pain signaling pathways. This disruption reduces the perception of pain and provides relief to the user.

### TAPS Session Procedures

Following enrollment, participants received detailed verbal and written instructions from the research team on the operation and use of the self-administered TAPS audio-based therapy system. The orientation included instructions on accessing the therapy sessions, proper use of the device, the recommended frequency and duration of use during the study period, and an opportunity to ask questions before beginning the intervention. To promote adherence, multiple reminders and follow-up contacts were implemented, including email and text message reminders from the research team. During the informed consent process, participants were informed of the potential risks and benefits associated with the intervention, including that no known risks had been identified and that potential benefits could include reductions in symptoms and pain, improved sleep quality, and enhanced overall well-being. Because the study was implemented within an active police workforce, self-administration was designed to accommodate varying shifts and operational responsibilities.

### Intervention Implementation and Outcome Assessment

Each participant was assigned a flexible 30-day TAPS intervention period, during which sessions were self-administered either as needed or at the participant’s convenience to accommodate police work schedules and operational responsibilities. Because the intervention was integrated into routine occupational duties, session timing and exposure could vary across participants, and completion of the full study across all 24 participants required slightly more than 5 months because of duty rotations, scheduling conflicts, illness, vacation, reassignment, attrition, and minor technical or operational issues. Session-level adherence and cumulative intervention exposure were not systematically captured through automated logs or complete participant diaries; therefore, the number of sessions completed by each participant, the total duration of use, and dose-response patterns could not be quantified.

Outcomes were exploratory and based on participant self-report, including condition improvement, symptom reduction, perceived pain reduction, perceived effectiveness, satisfaction, safety and tolerability, and likelihood of continued use. These outcomes were not measured using validated pre-post clinical instruments, and no predefined threshold for clinically meaningful improvement was applied; therefore, findings were interpreted as participant-reported perceptions rather than evidence of clinical efficacy. Adverse events were assessed through participant reporting during the study period; however, the pilot study did not include a formal, prospectively defined adverse event surveillance protocol, so the absence of reported adverse events should not be interpreted as establishing intervention safety.

### Statistical Analysis

We used percentages as measures of effect and the chi-square test and Fisher exact test for significance testing. All *P* values were 2 sided, with a level of significance of .05. Specifically, we explored symptom reduction, satisfaction, and usability within this small pilot study sample. We conducted nonparametric tests to explore the statistical significance of the relationships between condition improvement, perceived effectiveness in symptom and pain relief, and overall satisfaction. A total of 26 participants were enrolled in the study. However, 2 (7.69%) participants had missing data on multiple study variables and were excluded from the analyses. Therefore, the final analytic sample consisted of 24 participants with complete information. CIs for the estimates were calculated using the Wilson score method because of the small sample size.

## Results

A total of 70 police officers attended the study presentations; 46 (65.7%) expressed interest and completed informed consent, 26 (37.1%) participants enrolled, and 24 (34.3%) completed the 30-day intervention and were included in the final analyses; 2 (2.86%) participants were excluded due to missing data. Participants’ demographic characteristics were not collected.

Participants’ self-reported health conditions are summarized in Table 1. Approximately one-third (8/24, 33.3%) reported having 2 or more health conditions, while two-thirds (16/24, 66.7%) reported a single condition. The most prevalent were musculoskeletal and pain conditions, including musculoskeletal disorders, injury, back pain, neck pain, joint pain, arthritis, and neuropathic pain, which were reported by 23 (88.5%, 95% CI 71-96) participants. Less commonly reported conditions included other chronic conditions (n=4, 15.4%, 95% CI 6.2-33.5); stress, anxiety, or depression (n=3, 11.5%, 95% CI 4-29); and sleep disorders (n=3, 11.5%, 95% CI 4-29). Headaches (n=2, 7.7%, 95% CI 2.1-24.1) and tendinitis or related soft tissue disorders (n=2, 7.7%, 95% CI 2.1-24.1) were reported less frequently. Most (n=20, 83.3%) participants expressed a strong interest in using the device.

**Table 1:** Self-reported health conditions among study participants (N=24).

Health conditions	Participants^a^, n (%; 95% CI^b^)
One health condition	16 (66.7; 46.7-82.0)
Two or more health conditions	8 (33.3; 18.0-53.3)
Musculoskeletal and pain conditions (including musculoskeletal disorders, injury, back pain, neck pain, joint pain, arthritis, and neuropathic pain)	23 (95.8; 79.8-99.3)
Other chronic conditions	4 (16.7; 6.7-35.9)
Stress, anxiety, or depression	3 (12.5; 4.3-31.0)
Sleep disorders	3 (12.5; 4.3-31.0)
Headaches	2 (8.3; 2.3-25.8)
Tendinitis and related soft tissue disorders	2 (8.3; 2.3-25.8)

^a^Participants could report more than 1 condition; therefore, percentages sum to more than 100%.

^b^CIs were calculated using the Wilson score method due to the small sample size.

Table 2 summarizes participants’ responses regarding health condition improvement, symptom reduction, perceived effectiveness of the audio therapy technology, and overall satisfaction. Following the audio therapy, 37.5% (9/24, 95% CI 18.8-59.4) of participants reported improvement in their condition, while 50% (12/24, 95% CI 29.1-70.9) reported symptom reduction and a significant decrease in pain intensity. Overall, 11 (45.8%, 95% CI 25.6-67.2) of 24 participants perceived the audio therapy technology to be effective. Most (14/24, 58.3%, 95% CI 38.8-75.5) participants reported neutral satisfaction, whereas 33% (8/24, 95% CI 15.6-55.3) indicated that they were either satisfied or very satisfied with the audio therapy.

**Table 2:** Participant responses related to health condition improvement and satisfaction following audio therapy (N=24).

Responses	Participants, n (%; 95% CI^a^)
Reported condition improvement	9 (37.5; 18.8-59.4)
Reported symptom reduction and reduced pain intensity	12 (50; 29.1-70.9)
Perceived technology as effective	11 (45.8; 25.6-67.2)
Neutral satisfaction	14 (58.3; 38.8-75.5)
Satisfied or very satisfied	8 (33.3; 15.6-55.3)

^a^CIs were calculated using the Wilson score method due to the small sample size.

Table 3 presents the associations between perceived technology effectiveness, participant satisfaction, and reported health condition improvement. Among the 11 participants who perceived the audio therapy technology as effective, 9 (81.8%, 95% CI 52.3-94.9) reported improvement in their condition, whereas only 2 (18.2%, 95% CI 5.1-47.7) did not report improvement (Fisher exact test, *P*<.001). Similarly, among the 12 participants who reported symptom reduction, 9 (75%, 95% CI 46.8-91.1) experienced condition improvement, while 3 (25%, 95% CI 8.9-53.2) did not (*P*<.001).

**Table 3:** Associations between perceived effectiveness, satisfaction, and health condition improvement.

Outcomes	Improved, n (%; 95% CI)	No improvement, n (%; 95% CI)	*P* value^a^
Perceived technology as effective (n=11)	9 (81.8; 52.3-94.9)	2 (18.2; 5.1-47.7)	<.001
Reported symptom reduction (n=12)	9 (75; 46.8-91.1)	3 (25; 8.9-53.2)	<.001
Satisfied or very satisfied (n=8)	6 (75; 40.9-92.9)	2 (25; 7.1-59.1)	.006
Neutral satisfaction (n=14)	2 (14.3; 4-39.9)	12 (85.7; 60.1-96)	<.001^b^

^a^Only selected response categories are displayed. *P* values were calculated using 2-sided Fisher exact tests based on all response categories for each outcome.

^b^The *P* value for satisfaction represents the overall association between the levels of the satisfaction variable and improvement status.

Satisfaction with the audio therapy system was also associated with reported improvement (*P*=.006). Of the 8 participants who were satisfied or very satisfied with the system, 6 (75%, 95% CI 40.9-92.9) reported condition improvement, whereas 2 (25%, 95% CI 7.1-59.1) did not. In contrast, among the 14 participants who reported neutral satisfaction, only 2 (14.3%, 95% CI 4-39.9) experienced improvement, whereas 12 (85.7%, 95% CI 60.1-96) reported no improvement.

Minor technical, operational, scheduling, and personnel-related issues were encountered during study implementation but were not documented in sufficient detail for formal analysis. No participant-reported adverse events were documented during the study; however, because a formal adverse event surveillance protocol was not implemented, the absence of reported adverse events should not be interpreted as evidence of intervention safety.

## Discussion

### Principal Findings

In this exploratory pilot case series of 24 police personnel, participants reported generally favorable experiences with a self-administered TAPS intervention implemented within an active occupational setting. More than one-third (n=9, 37.5%) of participants experienced improvement in their condition, and half (n=12, 50%) reported reduced symptoms, including notable perceived pain reduction. Satisfaction ratings were largely neutral to positive, with one-third (n=8, 33.3%) reporting satisfaction or being highly satisfied. Interest in using the device was also high, with 83.3% (n=20) of participants expressing strong interest. These findings provide preliminary information regarding participant acceptability and self-reported experiences with the intervention; however, they should not be interpreted as evidence of clinical efficacy.

The findings observed for condition improvement, perceived effectiveness, and satisfaction should also be interpreted cautiously. Participants who reported condition improvement were more likely to characterize the intervention as effective, and satisfaction differed according to improvement status. However, these outcomes are conceptually related and were based on participant self-report. Thus, the observed findings may reflect a clustering of favorable perceptions rather than independent evidence of treatment benefit. The small sample size further limits the stability and interpretability of these estimates. Accordingly, these results should be considered hypothesis generating and may help inform the selection of design features for future analytic studies designed a priori to test the hypothesis [15].

Participants with and without preexisting conditions expressed high interest, suggesting potential broad appeal. In a cross-sectional study, more than 90% of patients seeking care in the emergency department (ED) expressed willingness to try nonpharmacological treatments for musculoskeletal pain [16]. This may have been due, at least in part, to the feasibility and flexibility that nonpharmacologic options, such as audio-based therapy, offer, as they can often be used outside of traditional clinical settings [16]. Nevertheless, the high level of baseline interest observed in this study warrants careful consideration. Strong preintervention interest may indicate acceptability, but it may also increase susceptibility to expectancy effects. Patients experiencing greater fatigue or pain-related interference with daily activities particularly expressed higher interest in psychosocial treatments, such as music therapy, likely because such treatments require minimal physical effort [16]. In another study, the expectation of obtaining pain relief enhanced the effectiveness of music therapy itself [10]. Therefore, the high baseline interest in the present sample should not be interpreted as evidence supporting therapeutic effectiveness. Rather, it highlights the importance of incorporating expectancy measures and appropriate comparator conditions in future studies.

The proportion of participants reporting improvement or symptom reduction supports the potential therapeutic value of multiwaveform sound-based interventions. Additionally, reduction in pain intensity aligns with existing evidence showing associations between auditory stimulation and the modulation of pain perception. In patients with chronic pain, theta-rhythm binaural beats were found to significantly decrease pain intensity and reduce everyday analgesic use [17]. Clinical pilot evidence also showed that low-frequency sound wave stimulation was effective in reducing pain and improving functional ability in patients with chronic back pain [18]. Our finding that all participants who reported improvement also perceived the technology as effective and safe suggests concordance between perceived benefit and user evaluation and may support long-term engagement with sound therapy.

### Strengths and Limitations

This pilot study has several strengths. It provides preliminary information regarding the implementation of a self-administered intervention within a real-world occupational setting characterized by variable schedules and operational demands. The inclusion of police personnel offers insight into intervention delivery in a workforce for whom fixed participation schedules may be difficult to maintain. The study also captured direct participant feedback regarding improvement, perceived effectiveness, satisfaction, and continued interest, which may help refine intervention procedures and outcome selection for future research.

Several major limitations should be considered when interpreting the findings of this study. First, because the study was descriptive and conducted without a comparison group, any observed changes should be considered hypothesis generating rather than evidence of intervention effectiveness. Second, the study relied predominantly on subjective participant-reported outcomes. Measures such as condition improvement, symptom reduction, perceived pain reduction, perceived effectiveness, and satisfaction may capture meaningful participant experiences but are susceptible to recall bias, social desirability bias, and expectation bias. Moreover, these outcomes were not predefined using standardized or validated clinical criteria and were based solely on participants’ self-reported perceptions collected through the study questionnaire. Accordingly, they should be interpreted as subjective perceptions rather than objective measures of clinical improvement or treatment efficacy. Third, the enrolled sample was clinically heterogeneous. Participants varied in the presence and type of baseline conditions and symptoms, limiting the ability to attribute findings to any single clinical population. The small sample size also precluded meaningful condition-specific analyses. Fourth, intervention exposure was flexible and self-administered. Although participants reported the number of sessions completed, session frequency, duration, cumulative intervention exposure, adherence, and formal protocol deviations were not systematically recorded. Consequently, these data do not permit a formal assessment of intervention feasibility and should be interpreted as implementation characteristics rather than formal feasibility outcomes. Likewise, variation in the number, timing, and duration of sessions may have influenced participant experiences and precluded evaluation of dose-response relationships. These findings nevertheless inform the design of future analytic studies, which should prospectively capture intervention adherence, session frequency, duration, cumulative exposure, protocol deviations, and reasons for nonadherence using predefined feasibility metrics. Fifth, any observed changes in condition improvement, perceived effectiveness, and satisfaction should be viewed as contributing to hypothesis generation. These constructs are conceptually overlapping, and participants with an overall favorable impression of the intervention may have responded positively across multiple related questionnaire items. Finally, the study was conducted in a single occupational setting and included police personnel aged 21 to 65 years. Occupational culture, work demands, participant expectations, and patterns of symptom reporting may differ from those of other populations. These factors limit the generalizability of the findings to broader clinical and community populations.

### Conclusions

This exploratory pilot study provides preliminary information regarding the implementation and acceptability of a self-administered TAPS intervention among police personnel. Although participants reported generally favorable experiences, the small sample size, the inclusion of participants with heterogeneous baseline conditions, and the reliance on self-reported outcomes should be considered when designing future studies. Future studies should incorporate clearly defined eligibility criteria, validated pre-post outcome measures, standardized intervention dosing, objective adherence monitoring, and systematic adverse event surveillance to test the hypothesis of whether the intervention provides clinically meaningful benefit.

## Abbreviations

ANS: autonomic nervous system
ED: emergency department
SSW: Scientific Sound Works
STROBE: Strengthening the Reporting of Observational Studies in Epidemiology
TAPS: Transdermal Acoustic Pain Suppression
